# Shift of Bacterial Community in Synanthropic Mite *Tyrophagus putrescentiae* Induced by *Fusarium* Fungal Diet

**DOI:** 10.1371/journal.pone.0048429

**Published:** 2012-10-31

**Authors:** Jan Hubert, Marta Nesvorná, Markéta Ságová-Marečková, Jan Kopecký

**Affiliations:** 1 Department of Pest Control of Stored Products and Food Safety, Crop Research Institute, Prague, Czech Republic; 2 Laboratory for Diagnostics and Epidemiology of Microorganisms, Crop Research Institute, Prague, Czech Republic; University of Waterloo, Canada

## Abstract

**Background:**

*Tyrophagus putrescentiae* (Acari: Astigmata) and *Fusarium* sp. co-occur in poorly managed grain. In a laboratory experiment, mite grazing resulted in significant reduction of fungal mycelium on cultivation plates. The destruction of mycelium appeared to be a result of an interaction between the mites, fungi and associated bacteria.

**Methodology and Principal Findings:**

A laboratory experiment was performed to simulate a situation of grain multiinfested by mites and *Fusarium* fungi. Changes of mite-associated bacterial community in *T. putrescentiae* were described in 3 habitats: (i) *T. putrescentiae* mites from a rearing diet prior to their transfer to fungal diet; (ii) fungal mycelium before mite introduction; (iii) mites after 7 day diet of each *Fusarium avenaceum*, *F. culmorum*, *F. poae* and *F. verticillioides*. Bacterial communities were characterized by 16 S rRNA gene sequencing. In total, 157 nearly full-length 16 S rRNA gene sequences from 9 samples representing selected habitats were analyzed. In the mites, the shift from rearing to fungal diet caused changes in mite associated bacterial community. A diverse bacterial community was associated with mites feeding on *F. avenaceum*, while feeding on the other three *Fusarium* spp. led to selection of a community dominated by *Bacillaceae*.

**Conclusions/Significance:**

The work demonstrated changes of bacterial community associated with *T. putrescentiae* after shift to fungal diets suggesting selection for *Bacillaceae* species known as chitinase producers, which might participate in the fungal mycelium hydrolysis.

## Introduction

It is believed that ancestors of synanthropic mites penetrated gradually from soil habitats to bird and rodent nests and switched from fungal diet to utilization of debris [1]. In the following step, they penetrated also to anthropogenic environments [2], where they colonized diversified habitats *i.e.* stored food, plant product, feed of domestic animals and pets, and house dust [3]. Fungivory seems to remain one of the feeding strategies of synanthropic mites, and may have important consequences to their successful survival in human habitats [4]. Such habitats contain plant debris, which are rich in carbohydrate sources but poor in nitrogen contents. Microorganisms growing on plant debris can supply the lacking nitrogen. Synanthropic mites are regarded as pests because they produce many compounds contaminating the indoor environment and cause allergic reaction in humans [5], [6]. The mites also interact and vector microorganisms of medical importance [7], [8].

*Tyrophagus putrescentiae* is one of the most common synanthropic mites with a cosmopolitan occurrence. Its feeding on fungi was reported in many studies and confirmed that *T. putrescentiae* is able to successfully develop on many fungal species [9], [10], [11]. The species frequently attacks the laboratory fungal cultures [12].

Potential for s utilization of bacteria as a food source for *T. putrescentiae* [13] was documented by lysozyme activity which was detected both in the whole body homogenates and fecal extracts of *T. putrescentiae* [14] and indicated digestive activity. Lysozyme hydrolyzes peptidoglycan (alternating β-(1,4) linked N-acetylglucosamine and N-acetylmuramic acid) in the cell wall of G+ bacteria; however, it can also cleave chitin (β-(1,4) linked polymer of N-acetylglucosamine).

In a laboratory experiment, fungal cultures grown on Petri dishes inoculated with *T. putrescentiae*, mite feeding resulted in total elimination of fungal mycelium [9]. In the previous experiments, we have observed the ability of *T. putrescentiae* to successfully develop on mycelium of *Fusarium avenaceum, F. culmorum, F. poae* and *F. verticillioides* [15] however, the switch in bacterial communities was described only by cultivation approach [16], [17], [18]. Although *Fusarium* spp. are known as “field fungi” infecting plants, these fungi secondarily continue to grow and produce mycotoxins in stored grain [19]. Poorly managed grain is often contaminated by mites including T. putrescentiae [20] and it is expected that T. putrescentiae interacts with fungi by yet unknown complex mechanisms.

In this study, we designed a model experiment simulating the situation in stored grain infested by mites and different *Fusarium* spp. strains. The aim of the study was to describe changes in mite-associated bacterial community induced by feeding on the cultures of *Fusarium* spp. and to find whether bacterial taxa able to participate in the elimination of the fungal mycelium are selected.

**Figure 1:**
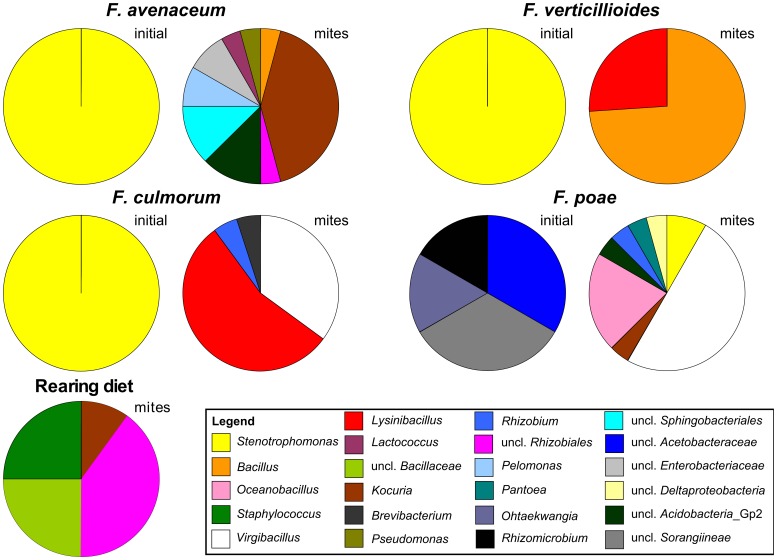
Phylogenetic assignment of bacterial 16 S rRNA gene sequences cloned from DNA extracted from *Tyrophagus putrescentiae* and *Fusarium* sp., cultures. Samples of *T. putrescentiae* shifted from rearing diet to *Fusarium* spp. diet are labelled as mites. The bacterial community associated with *Fusarium* culture before mite introduction is labelled as initial.

**Table 1:** The identification and the numbers of bacterial clones obtained from the samples; the classification reliability is given by confidence threshold (ct) value (RDP Classifier, [23]).

Order	Family	Genus	ct %	start	XFa	TFa	XFc	TFc	XFv	TFv	XFp	TFp
uncl. *Acidobacteria*_Gp2						3						1
*Actinomycetales*	*Brevibacteriaceae*	*Brevibacterium*	100					1				
	*Micrococcaceae*	*Kocuria*	93–100	2		10						1
*Sphingobacteriales*	uncl. *Sphingobacteriales*					3						
*Bacteroidetes inc. sedis*		*Ohtaekwangia*	100								1	
*Bacillales*	*Bacillaceae*	*Bacillus*	99–100			1				17		
		*Lysinibacillus*	100					11		6		
		*Oceanobacillus*	100									5
		*uncl. Bacillaceae*		5								
		*Virgibacillus*	81–89					7				12
	*Staphylococcaceae*	*Staphylococcus*	98–100	5								
*Lactobacillales*	*Streptococcaceae*	*Lactococcus*	100			1						
*Rhizobiales*	*Rhizobiaceae*	*Rhizobium*	100					1				1
	uncl. *Rhizobiales*			8		1						
*Alphaproteobacteria inc. sedis*		*Rhizomicrobium*	100								1	
*Rhodospirillales*	*Acetobacteraceae*	uncl. *Acetobacteraceae*									2	
*Burkholderiales*	*Comamonadaceae*	*Pelomonas*	100			2						
*Enterobacteriales*	*Enterobacteriaceae*	*Pantoea*	99									1
		uncl. *Enterobacteriaceae*				2						
*Pseudomonadales*	*Pseudomonadaceae*	*Pseudomonas*	84			1						
*Xanthomonadales*	*Xanthomonadaceae*	*Stenotrophomonas*	100		13		20		7			2
*Myxococcales*	uncl. *Sorangiineae*										2	
uncl. *Deltaproteobacteria*												1

Legend: Start - samples from *Tyrophagus putrescentiae* originating from the rearing diet before the transfer to the fungal diets; X – samples form fungal cultures before mite introduction; T – samples from *Tyrophagus putrescentiae* on fungal diet sampled 7 days after the introduction; Fa -*Fusarium avenaceum*, Fc - *F. culmorum*, Fp - *F. poae* and Fv - *F. verticillioides.*

## Materials and Methods

### Mites

Synanthropic mite *Tyrophagus putrescentiae* (Schrank) (Acari: Acarididae) originated from the laboratory cultures maintained at the Crop Research Institute, Praha, Czech Republic (CZ). Mites were reared on a mixture of oat flakes, wheat germs, and Pangamin - dried yeast extract (Rapeto, Bezdruzice, CZ) (ratio: 10∶10:1 wt). The diet was powdered and sieved and sterilized in a thermostat (70°C) for 0.5 h. About 1.5 g of the diet was placed into Iwaki tissue cell chambers (P-Lab, Praha, CZ) and chambers were maintained in Secador desiccators (P-Lab) at 85% relative humidity and 25±3°C in darkness. Mites were separated using Stemi 2000 C dissection microscope (C. Zeiss, Jena, Germany) from the plugs or surface of the chambers by brush. Mites were not sexed and aged.

**Figure 2:**
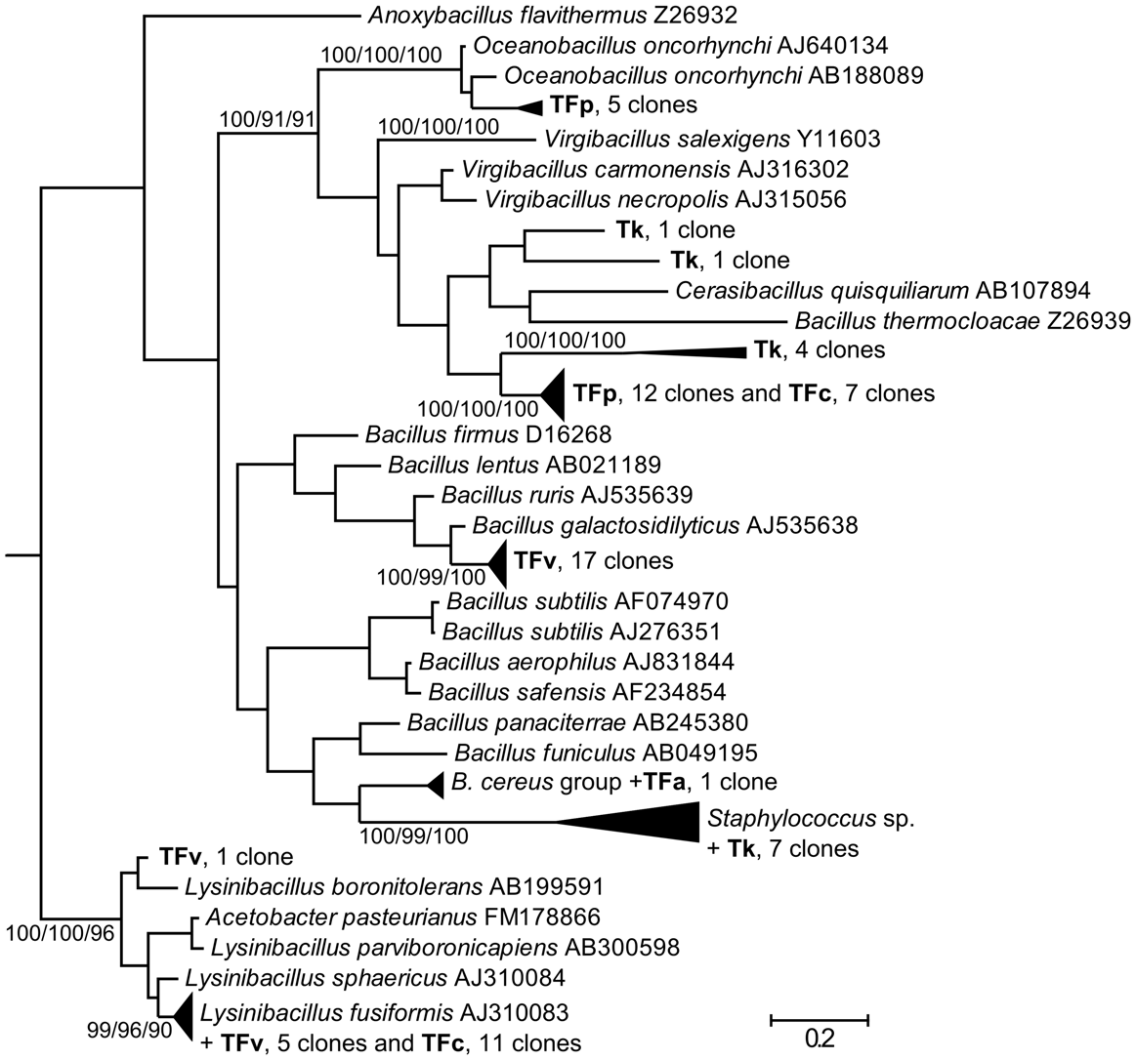
Phylogenetic analysis of clones and isolates belonging to *Bacillales* together with their closest neighbors available in RDP database. Phylogeny was inferred by Bayesian analysis of 16 S rRNA gene sequences of 69 clones from *Tyrophagus putrescentiae* grown on the tested diets (Tk - rearing diet, TFa -*Fusarium avenaceum*, TFc - *F. culmorum*, TFp - *F. poae* and TFv - *F. verticillioides*) and 39 selected type-strain sequences from the genera *Bacillus* and *Staphylococcus*. Branch lengths correspond to mean posterior estimates of evolutionary distances (scale bar, 0.2). Branch labels indicate the Bayesian posterior probability and for selected branches also supporting bootstrap values from maximum-likelihood and neighbor-joining analyses. The phylograms were outgrouped using the *E. coli* sequence U00096.

### *Fusarium* spp. strains

The following fungal strains were used in the experiment: *Fusarium poae* ((Peck) Wollenw. 1913); strain No 1 was isolated from barley harvested in Prague-Ruzyně (CZ) in August 2005, *F. culmorum* ((W. G. Sm.) Sacc. 1892) strain No 7 originated from damaged corn cobs obtained in Ivanovice na Hané (CZ) in September 2003, *F. verticillioides* ((Sacc.) Nirenberg 1976) strain No 8 was isolated from kernels of maize in Čáslav (Cz) in October 2006, and F. avenaceum ((Corda) Sacc. 1886) strain No 13 was isolated from poppies in Opava in August 2009. The obtained fungal cultures were maintained in laboratory cultures in the Crop Research Institute. One week before the experiment, the fungi were inoculated on oatmeal agar plates [21].

**Figure 3:**
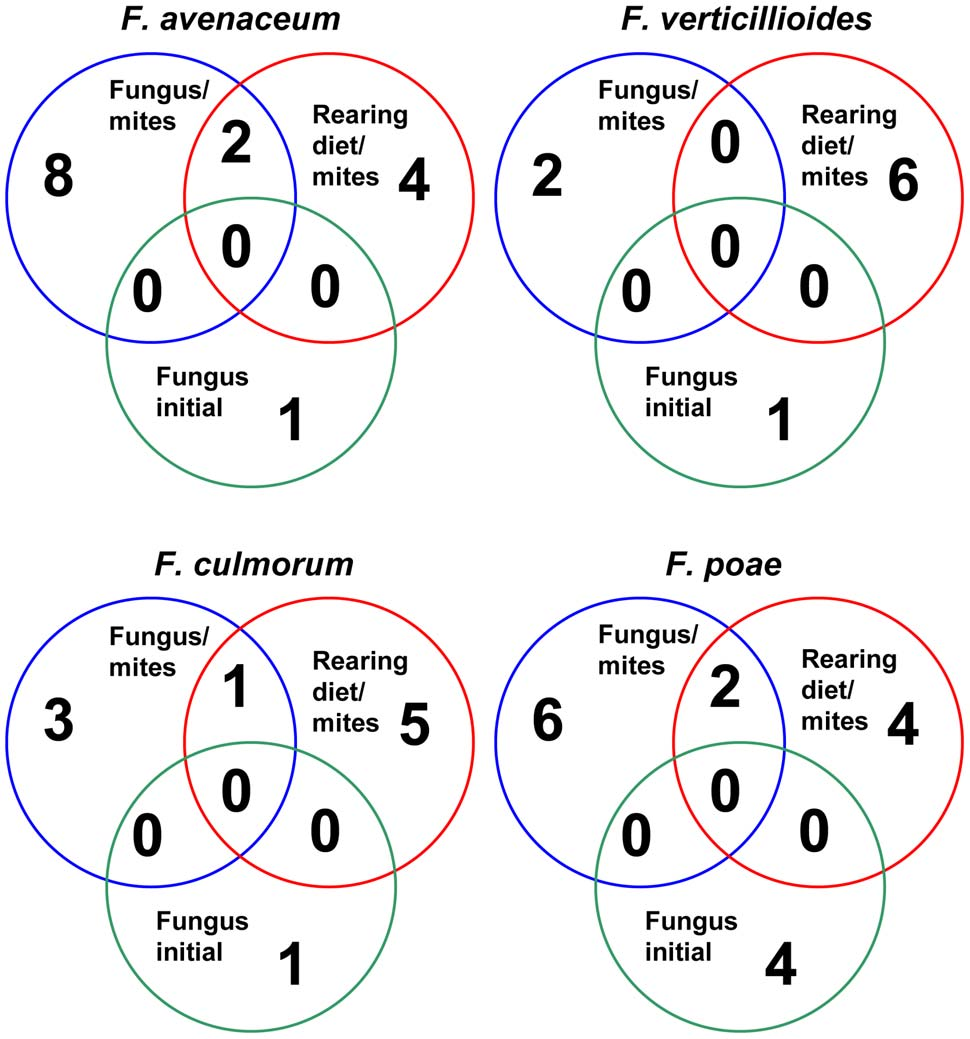
Comparison of bacterial communities based on operational taxonomic units (OTU) defined at distance 0.06 using Venn diagram. The numbers of OTU are presented separately for initial situation (fungus before mite introduction) and mites feeding on rearing diet and fungal diet.

### Experimental Design

Selected fungal species were cultivated on oatmeal agar in triplicate for 7 days. A mycelium sample of each species was used for DNA extraction. About 100 mites were added to every plate and incubated for 7 days at 25 °C. Samples of mites collected after incubation were surface-sterilized: (i) ethanol, bleach (4.7% aqueous NaClO), Tween 20 (ratio: 5∶5:0.01 v:v:v) –2 minutes; (ii) 96% ethanol –2 minutes; (iii) 3 times sterile redistilled water –2 minutes. The solution was removed by centrifugation (Espresso centrifuge, Thermo Scientific). Replicates were mixed to obtain one sample of mites per diet of one fungal species. As a reference, mites were also collected from the original rearing diet.

### 16S RNA Gene Cloning and Sequencing

The DNA extraction was done using Wizard® SV Genomic DNA Purification Kit (Promega, Madison, WI) according to manufacturer's instruction. Extracted DNA was stored in a freezer at −20°C before analyses. PCR amplification of 16 S rRNA gene was performed with universal bacterial primers – UF: 5′-AGA GTT TGA TYM TGGC 3′ (position 8–23) and UR: 5′-GYT ACC TTG TTA CGA CTT (position 1496–1514) [22] using C1000 thermal cycler (Bio-Rad, Hercules, CA, USA). A total volume of 25 µl PCR reaction mixture contained final concentration of 200 µM dNTPs, 3 mM MgCl_2_; forward and reverse primers (100 nM each), 0.5 unit *Taq* polymerase (all Promega, Madison, WI) and 300 ng template DNA (mixture of mite genomic DNA and bacterial DNA). Amplification conditions were as follows: Two min at 94°C followed by 30 cycles of 90 s at 94°C, 90 s at 50°C, and 60 s at 72°C, followed by final extension for 10 min at 72°C and 4°C hold [22]. Resulting PCR products were purified with GFX PCR DNA and Gel Band Purification Kit (Amersham Bioscience, UK) and cloned using pGEM®-T Easy Vector (Promega, Madison, WI, USA). Selected clones were sequenced in the Macrogen (Seoul, Korea). Nearly full length sequences were assembled with CodonCode Aligner, version 1.5.2 (CodonCode Corporation, Dedham, MA, USA) and assigned to bacterial taxonomy using Ribosomal database project naïve Bayesian rRNA classifier [23].

### Phylogenetic Analysis

An initial alignment of partial 16 S rRNA gene sequences was performed using Muscle 3.6 [24]. Phylogeny was inferred by Bayesian analysis using Markov-Chain Monte Carlo sampler in Phase 2.0 software [25]. Neighbor-Joining method based on the Jukes-Cantor distance matrix, (1000 bootstrap resamplings) in Phylip 3.68 package [26], and Maximum-likelihood method with Jukes-Cantor substitution model (1000 bootstrap resamplings) in Phyml software [27]. The resulting phylograms were finalized using MEGA version 4 [28].

## Results

### Bacterial Communities of Mites and Fungal Cultures Before their Co-incubation

A total of 46 nearly-full length sequences were obtained from samples of fungal species before mite introduction and 20 sequences were from mites of the original rearing diet. The sequences were deposited in GenBank under Accession Nos. JX001188 - JX001253.

Bacterial communities associated with *F. avenaceum*, *F. culmorum* and *F. verticillioides* were formed only by bacteria related to *Stenotrophomonas*. Bacterial community of *F. poae* was more diverse and included bacteria related to *Acetobacteraceae*, *Sphingobacteriales*, *Rhizobiales* and uncultured *Sorangiineae* (Table 1). The initial sample of bacterial community in mites originated from rearing diet showed a developed bacterial community. The most abundant were bacteria with high similarity to *Kocuria*, followed by uncultured *Bacillaceae*, *Virgibacillus*, *Staphylococcus* and *Bartonella*-like bacteria (uncl. *Rhizobiales*) (Figure 1).

### Bacterial Communities of Mites Feeding on Fugal Cultures

A total of 91 nearly-full length sequences of 16 S rRNA gene were obtained from the samples of mites grazing on fungal cultures for 7 days. The sequences are deposited in GenBank under Accession Nos. JX001254 - JX001344. The shift of diet caused by transfer of mites from rearing diet to the *Fusarium* spp. cultures changed associated bacteria (Figure 1). The changes of the bacterial community were influenced by the fungal species.

Bacteria related to *Kocuria* prevailed in the specimens originating from *F. avenaceum* diet reminding the original diet. However, the other detected bacteria including *Pelomonas*, *Lactococcus*, *Pseudomonas*, *Bacillus* and uncultured group 2 of *Acidobacteria* differed from the initial sample. In mites grown on *F. culmorum* diet, prevailing bacteria had high 16 S rRNA gene sequence similarity to *Lysinibacillus*. Those were followed by bacteria phylogenetically related to *Virgibacillus,* uncultured *Bacillaceae, Brevibacterium* and *Rhizobium*. Mites grown on *F. verticillioides* diet harboured bacterial community, which consisted namely of bacteria related to *Bacillus* and *Lysinibacillus* (Figure 2).

The most diversified bacterial community was associated with mites grown on *F. poae* diet, although part of the spectrum was similar to the initial stage, *i.e.* belonging to *Kocuria,* uncultured *Bacillaceae*, and *Virgibacillus*. The clones belonging to *Stenotrophomona*s corresponded to the situation of fungal culture without mites. The diet specific clones were related to *Oceanobacillus*, *Pantoea*, *Rhizobium,* uncultured *Deltaproteobacteria*, and *Acidobacteria* Group 2.

### Comparison of the Bacterial Communities

Venn diagrams (Figure 3) at distance 0.10 showed that bacterial taxa occurring in any of the fungal species before mite introduction were never identified in mite bodies. Differently, the taxa present in mite bodies before the shift of their diets still persist in mites after the shift. Mixed bacterial communities were formed in specimens feeding on *F. poae*, *F. culmorum* and *F. avenaceum* diets, containing newly emerging bacterial taxa and taxa, which had been present in control diets. A different situation occurred in mites feeding on *F. verticillioides* diet. After the shift from control to *F. verticillioides* diet, the change in bacterial taxa was substantial with no common taxa occurring in the control and persisting in *F. verticillioides* diet.

In conclusion, *Bacillaceae* clones were responsible for the differences in mite associated bacterial communities. The *Bacillaceae* clones were observed in control mites and those feeding on the fungal diets but not in the fungal cultures without mites. In particular, species-specific clusters of *Bacillaceae* clones were found in mites on a diet of *F. verticillioides* (17 clones related to *Bacillus galactosidilyticus*) and *F. poae* diet (5 clones related to *Oceanobacillus oncorhynchi*). The reaming two clusters included clones from either *F. verticillioides,* and *F. culmorum* (*Lysinibacter sp.*) diets, or *F. poae* and *F. culmorum* (*Bacillus* sp.) diets.

## Discussion

Changes of the bacterial community associated with *T. putrescentiae* after transfer to fungal diets were observed. The resulting community was enriched particularly with bacterial species, which may participate in hydrolysis of fungal mycelium. Such changes in mite-associated bacterial community were expected because it has been documented previously that shifts of insect dietary regimes led to selection of new intestinal microbial communities [29], [30]. In this study, mites not only developed different gut associated bacterial community but also introduced several bacterial taxa of *Bacillaceae*, which might contribute to destruction of fungal mycelium. The shift of mite-associated bacterial community was influenced by original status on control diet because some originally present bacterial taxa persisted in specimens feeding on *F. poae*, *F. culmorum* and *F. avenaceum* diets. However, in specimens feeding on *F. verticillioides* diet, the bacterial community changed completely.

In a previous study, we have described a diverse internal bacterial community of *T. putrescentiae*, which included genera of *Kocuria*, *Bacillus* and *Staphylococcus* [31]. These bacteria were identified both among the clones and isolates and their presence was confirmed in this study. *Bartonella*-like and *Pseudomonas, Virgibacillus* and uncultured *Bacillaceae* were found in *T. putrescentiae*, but were not detected previously [31].

Differences in occurrence of *Bacillaceae* clones were observed between control mites and those feeding on the fungal diets and *Bacillaceae* clones were absent in the fungal cultures without mites. It suggests that mites introduce mainly *Bacillaceae*, *i.e. Bacillus* sp., *Bacillus galactosidilyticus*, *Oceanobacillus oncorhynchi* and *Lysinibacter* sp. to the fungal cultures. These bacteria can interact with mites and fungi and also change the palatability of fungi to the mites [16], [18]. In a previous study, the suitability of *Fusarium* spp. fungal diet for *T. putrescentiae* development decreased in the order from *F. verticillioides, F. culmorum, F. avenaceum* to *F. poae* being the least suitable for *T. putrescentiae* development [15].

The introduction of *T. putrescentiae* to *Fusarium* sp. fungi led to the elimination of fungal mycelium [15]. Bacterial lysis of fungi has been documented *e.g.* in *Bacillus cereus* affecting *Fusarium oxysporum* possibly using chitinase and laminarinase activities. However, incubation of fungal mycelium with chitinase alone did not result in the fungus lysis [32]. That indicated that the process might be more complex.Production of chitin degrading enzymes was described in several *Bacillaceae* including *B. circulans* [33], *B. licheniformis* [34], *B. cereus* [35], *B. subtilis*, *B. pumilus* and *Virgibacillus marismortui* [36]. Although chitinase activity was not assayed in the present study, it had been documented previously for extracts of *T. putrescentiae* [18]. It is known that the ability to lyse fungal hyphae, rather than chitin only, may provide growth substrate for chitinolytic soil bacteria [37]. Our suggestion is that the lytic activity resulting in the degradation of fungal hyphae in our experiments was conferred mainly by *Bacillaceae*. Mite contribution was mainly in fragmentation of mycelium by chelicerae [15], [17] and dispersal of bacteria through the fungal culture by feces in a similar way as suggested for fungal spores [4], [8].

Mites can digest contents of fragmented and partly destroyed mycelium together with bacteria growing on fungus. Digestion of bacteria has been documented previously for *T. putrescentiae* [13]. Also, based on the observed shift of bacterial community to one dominated by *Bacillus galactosidilyticus* on specimens feeding on *F. verticillioides* diet it can be suggested that bacteria can be responsible for increased nutritional value of *F. verticillioides* diet for *T. putrescentiae.* Such mixed feeding strategy seems to be more beneficial than utilization of the fungus alone. Although the experiments were conducted in the laboratory, similar interactions can be expected in the habitats where mites and fungi co-occur naturally.

## References

[1] O’Connor BM (1979) Evolutionary origins of astigmatid mites inhabiting stored products. Recent Adv. Acarol. 1: 273–278.

[2] O’Connor BM (1982) Evolutionary Ecology of Astigmatid Mites. Ann. Rev. Entomol. 27: 385–409.

[3] Colloff MJ (2009). Dust mites. CSIRO Publishing, Collingwood, Victoria, Australia.

[4] Sinha RN (1979) Ecology of microflora in stored grain. Annales of Technical Agriculture 28: 191–109.

[5] Arlian LG, Vyszenski-Moher DL, Johansson SG, van Hage-Hamsten M (1997) Allergenic characterization of Tyrophagus putrescentiae using sera from occupationally exposed farmers. Ann. Allergy Asthma Immunol. 79: 525–529. 10.1016/S1081-1206(10)63060-8 9433368

[6] Jeong KY, Lee H, Lee JS, Lee J, Lee IY, et al (2007) Molecular cloning and the allergenic characterization of tropomyosin from *Tyrophagus putrescentiae*. Protein Pept. Lett. 14: 431–436. 10.2174/092986607780782777 17584167

[7] Armitage DM, George CL (1986) The effect of three species of mites upon fungal growth on wheat. Exp Appl Acarol. 2: 111–124. 10.1007/BF01213755 3451862

[8] Hubert J, Stejskal V, Munzbergová Z, Kubátová A, Vánová M, et al (2004) Mites and fungi in heavily infested stores in the Czech Republic. J Econ Entomol. 97: 2144–2153. 10.1093/jee/97.6.2144 15666776

[9] Smrz J, Catska V (1987) Food selection of the field population of *Tyrophagus putrescentiae* (Schrank) (Acari: Acaridia). J. Appl. Entomol. 104: 329–335.

[10] Smrz J, Catska V (1989) The effect of the consumption of some soil fungi on the internal microanatomy of the mite Tyrophagus putrescentiae (Schrank) (Acari: Acaridida). Acta Universitatis Carolinae-Biologica 33: 81–93.

[11] Hubert J, Jarosik V, Mourek J, Kubatova A, Zdarkova E (2004) Astigmatid mite growth and fungi preference (Acari: Acaridida): Comparisons in laboratory experiments. Pedobiologia 48: 205–214.

[12] Duek L, Kaufman G, Palevsky E, Berdicevsky I (2001) Mites in fungal cultures. Mycoses 44: 390–394. 11766104 10.1046/j.1439-0507.2001.00684.x

[13] Erban T, Hubert J (2008) Digestive function of lysozyme in synanthropic Acaridid mites enables utilization of bacteria as a food source. Exp. Appl. Acarol. 44: 199–212. 10.1007/s10493-008-9138-x 18357505

[14] Childs M, Bowman CE (1981) Lysozyme activity in six species of economically important astigmatid mites. Comp. Biochem. Physiol. Part B. 70: 615–617.

[15] Nesvorna M, Gabrielova L, Hubert J (2012) *Tyrophagus putrescentiae* is able to graze and develop on *Fusarium* fungi of mycotoxins importance under laboratory conditions. J. Stored Prod. Res. 48: 37–45.

[16] Smrz J, Svobodova J, Catska V (1991) Synergetic participation of *Tyrophagus putrescentiae* (Schrank) (Acari, Acaridida) and its associated bacteria on the destruction of some soil micromycetes. J. Appl. Entomol. 111: 206–210.

[17] Smrz J (2003) Microanatomical and biological aspects of bacterial associations in *Tyrophagus putrescentiae* (Acari: Acaridida). Exp. Appl. Acarol. 31: 105–113. 10.1023/b:appa.0000005111.05959.d6 14756405

[18] Smrz J, Catska V (2010) Mycophagous mites and their internal associated bacteria cooperate to digest chitin in soil. Symbiosis 52: 33–40.

[19] Magan N, Hope R, Cairns V, Aldred D (2003) Post-harvest fungal ecology: Impact of fungal growth and mycotoxin accumulation in stored grain. Eur. J. Plant Pathol. 109: 723–730.

[20] Athanassiou CG, Palyvos NE, Eliopoulos PA, Papadoulis GT (2001) Distribution and migration of insects and mites in flat storage containing wheat. Phytoparasitica. 29: 379–392.

[21] Samson RA, Hoekstra ES, Frisvad JC, Filtenborg O (1996). Introduction to foodborne fungi. Baarn & Delft.

[22] Barbieri E, Paster BJ, Hughes D, Zurek L, Moser DP, et al (2001) Phylogenetic characterization of epibiotic bacteria in the accessory nidamental gland and egg capsules of the squid *Loligo pealei* (Cephalopoda:Loliginidae). Environ. Microbiol. 3: 151–167. 10.1046/j.1462-2920.2001.00172.x 11321532

[23] Wang Q, Garrity GM, Tiedje JM, Cole JR (2007) Naïve Bayesian Classifier for Rapid Assignment of rRNA Sequences into the New Bacterial Taxonomy. Appl. Environ. Microbiol. 73: 5261–5267. 10.1128/AEM.00062-07 PMC1950982 17586664

[24] Edgar RC (2004) MUSCLE: multiple sequence alignment with high accuracy and high throughput. Nucleic Acids. Res. 32: 1792–1797. 10.1093/nar/gkh340 PMC390337 15034147

[25] Jow H, Hudelot C, Rattray M, Higgs P (2002) Bayesian phylogenetics using an RNA substitution model applied to early mammalian evolution. Mol. Biol. Evol. 19: 1591–1601. 10.1093/oxfordjournals.molbev.a004221 12200486

[26] Felsenstein J (1989) PHYLIP-Phylogeny inference package (version 3.2). Cladistics 5: 164–166.

[27] Guindon S, Gascuel O (2003) A simple, fast, and accurate algorithm to estimate large phylogenies by maximum likelihood. Syst. Biol. 52: 696–704. 10.1080/10635150390235520 14530136

[28] Tamura K, Dudley J, Nei M, Kumar S (2007) MEGA4: Molecular Evolutionary Genetics Analysis (MEGA) software version 4.0. Mol. Biol. Evol. 24: 1596–1599. 10.1093/molbev/msm092 17488738

[29] Dillon RJ, Dillon VM (2004) The gut bacteria of insects: Nonpathogenic Interactions, An. Rev. Entomol. 49: 1–71. 10.1146/annurev.ento.49.061802.123416 14651457

[30] Santo Domingo JW, Kaufman MG, Klug MJ, Holben WE, Harris D, et al (1998) Influence of diet on the structure and function of the bacterial hindgut community of crickets. Mol Ecol 7: 761–767.

[31] Hubert J, Kopecky J, Perotti AM, Nesvorna M, Braig HR, et al (2012) Detection and identification of species-specific bacteria associated with synanthropic mites. Microbial. Ecol. 63: 919–928. 10.1007/s00248-011-9969-6 22057398

[32] Mitchell R, Alexander M (1963) Lysis of soil fungi by bacteria. Can. J. Microbiol. 9: 169–177.

[33] Watanabe T, Oyanagi W, Suzuki K, Tanaka H (1990) Chitinase system *Bacillus circulans* WL-12 and importance of chitinase A1 in chitin degradation. J. Bacteriol. 172: 4017–4022. 10.1128/jb.172.7.4017-4022.1990 PMC213387 2361948

[34] Trachuck LA, Revina LP, Shemyakina TM, Chestukhina GG, Stepanov VM (1996) Chitinases of *Bacillus licheniformis* B6839: isolation and properties. Can. J. Microbiol. 42: 307–315.

[35] Pleban S, Chernin L, Shet I (1997) Chitinolytic activity of endophytic strain of *Bacillus cereus*. Lett. Appl. Microbiol. 25: 284–288. 10.1046/j.1472-765x.1997.00224.x 9351279

[36] Essghaier B, Fardeau ML, Cayol JL, Hajlaoui MR, Boudabous A, et al (2009) Biological control of grey mould in strawberry fruits by halophilic bacteria. J. Appl. Microbiol. 106: 833–846. 10.1111/j.1365-2672.2008.04053.x 19191973

[37] De Boer W, Klein-Gunnewiek PJA, Lafeber P, Janse JD, Spit BE, et al (1998) Antifungal properties of chitinolytic dune soil bacteria. Soil Biol. Biochem. 30: 193–203.

